# Measuring Patient Satisfaction and Factors Affecting it in the General Surgery Setting in Jeddah

**DOI:** 10.7759/cureus.6497

**Published:** 2019-12-28

**Authors:** Bassel A Almehman, Zaher Mikwar, Atheel Balkhy, Heyam Jabali, Bassam S Hariri, Nada Y Baatiah

**Affiliations:** 1 General Surgery, College of Medicine, King Saud Bin Abdulaziz University for Health Sciences, Jeddah, SAU; 2 Surgical Oncology, King Abdulaziz Medical City, Ministry of National Guard Health Affairs, Jeddah, SAU; 3 Medicine, College of Medicine, King Saud Bin Abdulaziz University for Health Sciences, Jeddah, SAU; 4 Surgery, College of Medicine, King Saud Bin Abdulaziz University for Health Sciences, Jeddah, SAU; 5 Clinical Nutrition, College of Applied Medical Sciences, King Saud Bin Abdulaziz University for Health Sciences, Jeddah, SAU

**Keywords:** saps, patient-centered care, patient satisfaction, general surgery clinics, assessing health-care, quality of care

## Abstract

Introduction

A patient-centered approach is critical to improving the overall quality of healthcare, and this also applies to the general surgery setting. To achieve this, it is important to assess patient satisfaction with healthcare, but this topic has not been investigated in the context of Jeddah. Therefore, the present study is the first one to assess patient satisfaction with care in the general surgery department and associated factors in Jeddah.

Methods

This was a descriptive, cross-sectional study that used a convenience sampling technique to select 307 patients from the outpatient clinic of the general surgery department at King Abdulaziz Medical City from November to December 2018. The cohort comprised 53.1% women and the age range was 18-70 years. For data collection, we used the Short Assessment of Patient Satisfaction questionnaire, which contains seven items related to the core domains of patient satisfaction. We modified this by adding two additional items reported in other studies. We also included age, gender, and level of education as variables in the analysis. The scores assigned to each item were compared based on gender, age, and level of education.

Results

The analysis showed that age, gender, or level of education did not affect the overall satisfaction level, and the majority of participants (93.8%) reported that they were satisfied or very satisfied. Specifically, the majority reported that they were satisfied or very satisfied with the treatment effect, the explanation provided by the clinician, and the care provided at the clinic. Further, the majority of them also felt that they had received a thorough examination and enough time with the consultant, that they felt respected by the healthcare provider and their concerns were heard and respected, and that they were encouraged to voice their feelings and concerns. The lowest score was related to whether the participants felt like their choices were considered when it came to making healthcare decisions. This could, therefore, be a potential area of improvement.

Conclusion

Overall, the current findings indicate that the practitioners in the current setting use a patient-centered, adult-to-adult approach to healthcare, and the patients are highly satisfied. One area of improvement could be medical decision-making, where patients’ preference could be given more consideration. Thus, these findings provide important insight into patient-centered care in this region.

## Introduction

Lately, institutions worldwide have been focusing on a more patient-centered approach to care, with the aim of improving the quality of healthcare. Assessing patient satisfaction is a vital part of this patient-centered care approach, but the assessment of patient satisfaction is limited by its subjective nature [1]. Nonetheless, patient satisfaction can be used to assess and improve healthcare services, compare different systems or institutions of care, and identify areas that require improvement [2-3]. Patient-centered care is also relevant to the clinical setting of general surgery. In order to ensure that patients undergoing treatment are provided with the best care, it is important to assess their satisfaction level during their visit to the clinic. In fact, assessing patient satisfaction is crucial for all parties involved in the healthcare system. It is not only important for institutions to highlight areas of improvement and compare different programs and departments as well as different institutions, but it is also critical for patients to know that their opinions and experiences are being taken into perspective.

Studies in other countries have shown that patient satisfaction reliably differs from the center where the study was conducted, or which population it studied [2]. To the best of our knowledge, we have not come across an article that assessed patient satisfaction in Saudi nationals. Furthermore, while several studies took into account the nonmodifiable factors of patient satisfaction [2-4], other studies only assessed the encounter between the patient and the physician without considering any patient-related information [1].

In this paper, we have attempted to assess patient satisfaction in order to identify areas of improvement in the outpatient clinics of the surgery department of King Abdulaziz Medical City in Jeddah. Since the emergence of patient-centered care, patient satisfaction questionnaires have become increasingly popular [1], and several questionnaire models have been constructed. Patient satisfaction is affected by a number of modifiable and non-modifiable factors [4], which were both explored by this study as it assessed the association of patient satisfaction with demographic features, education, employment, fluency in English, and health literacy [5]. Specifically, in this paper, we aimed to assess the current satisfaction level of patients during their interaction with physicians in surgery outpatient clinics, as well as to identify the factors affecting patients’ experience. To the best of our knowledge, this is the first such study to be conducted in this region, and therefore, the findings would be highly useful to determine what changes are needed in the local context to improve patients’ healthcare and satisfaction.

## Materials and methods

Study cohort

 This study received the approval of King Abdullah International Medical Research Center, Jeddah, Saudi Arabia, and the Institutional Review Board, numbered SP18/464/J. The study is a descriptive, cross-sectional one that uses a convenience sampling technique for the selection of the study population, in order to estimate the patients’ satisfaction with the general surgery clinics, to identify the factors that affect it, and to determine the level of care provided to the patients by physicians. The study location was King Abdulaziz Medical City in Jeddah, and it was conducted from November to December 2018. We included a total of 307 participants within an age range of 18-70 years and included both Saudi and non-Saudi nationals.

Study design

 We used a self-administered, close-ended questionnaire to collect the data. The questionnaires were designed and modified based on the Short Assessment of Patient Satisfaction (SAPS) questionnaire that was developed by Hawthorne et al. [1]. The SAPS questionnaire consists of seven items for assessing the core domains of patient satisfaction: treatment satisfaction, explanation of treatment results, clinician care, participation in medical decision-making, respect by the clinician, time with the clinician, and satisfaction with hospital/clinic care. In addition, we included two more questions that we thought were necessary but were not included in the SAPS questionnaire: whether the patient felt the physician was listening and paying attention to their comments and concerns, and whether and physician encouraged the patient to talk and express themselves. Each question had five responses that were scored from 0 to 4. Thus, the total score possible was 36. The scores were interpreted as follows: 0-13 = very dissatisfied, 14-23 = dissatisfied, 24-33 = satisfied, 34-36 = very satisfied. Three demographic items were also added: age, gender, and level of education. The questionnaires were provided in both English and Arabic. SAPS reliability analysis of the modified questionnaire yielded a Cronbach’s alpha α value of 0.77, which is indicative of good reliability. 

 All participants were required to sign an informed consent form before responding to the questionnaire, and no identifying information was collected.

Data analysis

 We entered all the data into a Microsoft Office Excel 2017 file and analyzed the data using the Statistical Package for Social Sciences (version 23.0). For descriptive statistics, we used proportions for qualitative data and median with interquartile range (IQR) for quantitative data. For assessing the relationship between patient satisfaction and the variables, we used the Mann-Whitney U-test and Kruskal-Wallis test.

## Results

A total of 307 patients who met the inclusion criteria were enrolled in the study, and 53.1% were female. The participants were assigned to one of six age categories: 18.9%, 18-30 years; 16.3%, 31-40 years; 21.8%, 41-50 years; 21.8%, 51-60 years; 12.7%, 61-70 years; and 8.5%, >70 years. With regard to the level of education, 37.8% had a college degree, 25.4% had no formal education, 20.8% had a high school degree, 13.4% had a middle school degree, and 2.6% had a higher education degree.

 The median patient satisfaction score was 33 (IQR = 6), with the least score being 8 and the maximum being 36. According to their scores, 1.6% (5) were very dissatisfied, 4.6% (14) were dissatisfied, 47.9% (147) were satisfied, and 45.9% (141) were very satisfied. No significant difference was found between the level of satisfaction according to gender (p = 0.17), age (p = 0.12), or level of education (p = 0.83). For the rest of the scores, please refer to Table 1.

**Table 1:** Patients' satisfaction scores.

	Very dissatisfied	Dissatisfied	Neither satisfied nor dissatisfied	Satisfied	Very satisfied
How satisfied are you with the effect of your {treatment/care}?	1.3% (4)	1.0% (3)	8.5% (26)	20.2% (62)	69.1% (212)
How satisfied are you with the explanations the {doctor/other health professional} has given you about the results of your {treatment/care}?	1.6% (5)	1.3% (4)	3.9% (12)	15% (46)	78.2% (240)
Are you satisfied with the care you received in the {hospital/clinic}?	3.6% (11)	2.0% (6)	5.5%(17)	20.8% (64)	68.1% (209)
	Strongly disagree	Disagree	Not sure	Agree	Strongly agree
The {doctor/other health professional} was very careful to check everything when examining you.	3.6% (11)	2.0% (6)	5.2% (16)	15.0% (46)	74.3% (228)
How satisfied were you with the choices you had in decisions affecting your health care?	19.2% (59)	6.2% (19)	6.5% (16)	16.0% (49)	52.1% (160)
How much of the time did you feel respected by the {doctor/other health professional}?	0.7% (2)	1.3% (4)	0.7% (2)	4.6% (14)	92.8% (285)
The time you had with the {doctor/other health professional} was sufficient.	2.9% (9)	0.3% (1)	3.3% (10)	15.0% (46)	78.5% (241)
You often felt that the care provider was listening and paying attention to your comments and concerns.	2.0% (6)	0.3% (1)	2.9% (9)	15.0% (46)	79.2% (243)
The care provider often encouraged you to talk and express yourself.	6.8% (21)	2.0% (6)	2.6% (8)	16.3% (50)	72.3% (222)

## Discussion

In this paper, we have assessed patient’s satisfaction with the care provided at King Abdulaziz Medical City, Jeddah, and also tried to determine which factors affect patient-centered care at this institution. These findings are being reported for the first time in this region and may form an important basis for identifying the areas of improvement in healthcare in this setting.

Previously, it has been proposed that demographic features such as age, gender, and level of education might play a role in the overall satisfaction of patients. Therefore, our study took these factors into consideration, but we found that there was no significant difference in the level of satisfaction based on age, gender, or level of education. This was inconsistent with the findings of a study conducted in Greater London and Essex by Ann Bowling, in which female gender and older age were found to be associated with higher satisfaction levels in a hospital outpatient clinic setting [6]. The study established that, contrary to general belief, older age groups had higher satisfaction levels due to the belief that their expectations were being met; in contrast, their younger counterparts had lower expectations and, therefore, reported lower satisfaction levels [6]. In another study, it was found that patients with higher levels of education had lower satisfaction levels: this finding could probably be explained by their higher expectations from healthcare [2]. In addition, this group of patients may have more experience with high-quality healthcare, and may, therefore, have been disappointed with the care provided [2]. In the current cohort, we have not found statistical significance regarding patients’ demographics and level of satisfaction, as mentioned above. Although we do not have a clear explanation of this, it could be hypothesized that demographics did not make a difference because patients felt they were listened to and respected and had sufficient time with the care provider regardless of their age or level of education.

 In the current study, the majority of participants were satisfied or very satisfied with their overall experience at the clinic. A high percentage (92.8%) felt that they were respected by the doctor/physician throughout the time of the consultation. This is especially important in building a good doctor-patient relationship, as it might contribute to better compliance and, thus, better clinical outcomes and patient satisfaction. This might also be related to the finding that, in our setting, 69.1% were very satisfied and 20.2% were satisfied with their clinical outcome, and only 1.3% and 1% were very dissatisfied and dissatisfied, respectively. Thus, it indicates that good clinical outcomes can contribute to a higher level of satisfaction of the patient.

 An adult-to-adult approach has favorable outcomes in the clinical setting. This can be achieved by listening to the patient, taking their thoughts and preferences into consideration, and making sure they are aware of their condition. Based on our findings, it seems that such an approach was also adopted in the current setting: 89.3% of the participants were satisfied or very satisfied with the explanations provided to them by their healthcare provider or physician about the result of their care/treatment. Additionally, 79.2% responded that they were very satisfied and 15% responded that they were satisfied when asked if they felt that their care provider was listening and paying attention to their comments and concerns. Further, 72.3% felt very encouraged by the care provider to talk and express themselves, while 6.8% felt that they were not encouraged to share and express themselves. Although 72.3% does not seem like a very high percentage, it is relatively high compared to the satisfaction scores for the other questions. The highest dissatisfaction was reported with regard to the patients’ choices in decisions affecting their health. Thus, this is a potential area for improvement that should surely be addressed, even though it is possible that in some clinical cases there is only one treatment plan for the patient, and therefore the patient has not much of a choice in decisions affecting their health.

 When asked whether they thought that the physician had performed a thorough examination, 5.2% stated that they were unsure, 2.0% were dissatisfied, and 3.6% were very dissatisfied with the examinations performed. These scores are relatively high compared to those for the other questions. However, it is beyond the scope of this study to investigate whether the physicians actually performed complete examinations in these cases but were mistakenly or incorrectly assessed by the participants. It is possible that the patients were not aware of what examinations are considered relevant to their condition and, therefore, believed that they were not thoroughly examined.

 There are certain limitations of this study that must be acknowledged. First, it is not clear whether the surveys were conducted before surgery, after surgery, or in the dressing clinics. The waiting times after the procedure were not considered, although this may affect satisfaction levels. Finally, patients’ expectations were not assessed, although this might also have provided important insight into whether higher satisfaction was reported when their expectations were met. Despite its limitations, this study provides important insights into patients’ satisfaction with healthcare and the factors associated with it in the context of Jeddah. These findings provide an important basis for more investigation into patient-centered care in this region and ways of improving care.

## Conclusions

Overall, the current findings indicate that the practitioners in the current setting use a patient-centered, adult-to-adult approach to healthcare, and the patients are highly satisfied. One area of improvement could be medical decision-making, where patients’ preferences could be given more consideration. Thus, these findings provide important insight into patient-centered care in this region.
